# Mechanisms for Auditory Perception: A Neurocognitive Study of Second Language Learning of Mandarin Chinese

**DOI:** 10.3390/brainsci9060139

**Published:** 2019-06-17

**Authors:** Jing Yang, Ping Li

**Affiliations:** 1 Center for Linguistics and Applied Linguistics, Guangdong University of Foreign Studies, Guangzhou 510420, China; yangjing@gdufs.edu.cn; 2 Bilingual Cognition and Development Lab, Guangdong University of Foreign Studies, Guangzhou 510420, China; 3 Department of Psychology and Center for Brain, Behavior, and Cognition, Pennsylvania State University, University Park, PA 16802, USA

**Keywords:** auditory perception, second language word learning, individual differences, functional magnetic resonance imaging, effective connectivity

## Abstract

Speech perception is an important early skill for language learning. This study uses functional magnetic resonance imaging (fMRI) to examine the relationship between auditory perception abilities and second language (L2) vocabulary learning in an effort to explore behavior-brain correlations. Twenty-one English monolinguals learned 48 auditory Chinese pseudowords over six weeks. Their pre-training abilities in non-linguistic pitch and linguistic tone perception significantly and positively predicted their novel word-learning performance, which correlated with their brain response patterns in the left Heschl’s gyrus. Analyses of regions of interest (ROIs) showed coactivation of the frontal and temporal regions during novel lexical retrieval, and the non-linguistic pitch perception ability modulated brain activations in these regions. Effective connectivity analyses further indicated a collaboration of a ventral stream for speech perception and a dorsal stream for sensory-motor mapping in the L2 network. The ventral stream, compared with the dorsal stream, played a more dominant role in auditory word learning as the L2 proficiency increased. Better pitch and tone perception abilities strengthened the ventral pathways and decreased the reliance on frontal regions. These findings are discussed in light of current models of speech processing and L2 learning.

## 1. Introduction

An essential skill of language learning is the decoding of speech sounds for the understanding of the meaning of these acoustic signals. Auditory perception abilities are critical for seamless integration of phonology, semantics, orthography, syntax, and pragmatics in the speech comprehension process. Auditory perception may be a vital factor underlying typical and atypical language development and speech learning across the lifespan [1,2,3,4,5]. However, the neural correlates underlying the relationship between auditory perception and second language (L2) learning remain unclear.

Recent years have witnessed a growing interest in the understanding of neurocognitive mechanisms of L2 learning. Many studies have revealed neurocognitive and neuroanatomical changes in the brain [6], in at least the following domains of learning: speech sounds [7,8,9,10,11], vocabulary [12,13,14,15,16], and morphosyntactic rules [17,18,19,20]. Several studies have been conducted with lab-based training [5,16,17], while others in classroom settings [18,19,20,21,22,23]. Further, some studies have linked the neurocognitive patterns to language learning success, showing differences in brain activation and connectivity patterns for successful versus less successful learners [10,24,25,26,27,28]. A few studies also attempted to identify whether individual differences in cognitive abilities (e.g., working memory, intelligence) might predict L2 learning success [18,29,30]. 

Up to now, there are only a few neuroimaging studies that have examined the relationship between auditory perception abilities and L2 word learning. These studies suggest that learning-related functional or structural brain changes in the speech-motor control and auditory-perceptual areas, such as the left superior temporal gyrus (STG) and inferior frontal gyrus (IFG), may be responsible for native and non-native sound processing [15,21,31,32,33,34]. For example, Wang et al. [11] reported an increase in the spatial extent of brain activation in the left STG with participants who received a two-week training in Mandarin tone identification. Similarly, Golestani and Zatorre [32] found that white matter connectivity patterns in the left Heschl’s gyrus and parietal lobe predicted phonetic learning success of the Hindi dental-retroflex contrasts. The pilot work of Wong et al. [15] on L2 lexical learning reported more brain activations in the left posterior superior temporal gyrus (pSTG) in successful learners compared with less successful learners following auditory word learning. Interestingly, the same group difference was observed even before the training. More recently, in an fMRI investigation of language aptitude for pronunciation and L2 learning, Hu et al. [33] found that phonetic coding ability predicted L2 pronunciation aptitude in advanced learners, and was correlated with neural responses in speech-motor control and auditory-perceptual areas, including the left IFG, premotor areas, anterior superior temporal gyrus (aSTG), and pSTG. 

As one of the few fMRI studies focusing on speech perception and L2, Qi et al. [28] reported a longitudinal fMRI study examining speech processing and its relationship with L2 learning. In this study, learners of Mandarin, after four weeks of intensive classes, showed increased activations in the left IFG and superior parietal lobule (SPL) in a Mandarin tone discrimination task, compared with their brain activations before the training. Learners’ performance was also associated with reduced activations in the right IFG but increased connectivity between the right IFG and left IFG, and between the right IFG and the left SPL. The authors found that models based on the pre-training brain activations served as a better predictor for L2 learning performance than the models based on the pre-training tone discrimination accuracy. However, Qi et al. did not examine participants’ specific auditory skill, and it is unclear whether their study implicated a relationship between lexical tone discrimination performance and L2 learning attainment. 

In the present study, to reveal the neurocognitive mechanism for auditory perception and L2 lexical learning, we extended our previous fMRI study of L2 learning [16] by focusing on the role of auditory perception abilities in the same group of learners. First, participants were asked to complete a battery of behavioral tasks measuring their perception abilities of non-linguistic pitch (pitch), linguistic tone (tone), onset (consonant), rhythm, and intonation. In this way, we were able to examine the distinct contributions of auditory perception abilities. Second, participants learned 48 auditory words with lexical tones in Mandarin Chinese, which differ from simple phonetic training, but reflect word learning, given that tones convey different semantic meanings in Mandarin Chinese. For the participants whose native language is English, tones only indicate non-linguistic pitch variations before training but would become linguistically meaningful after training. After 18 30-min training sessions over six weeks, participants completed a sound-picture association judgment task when their brain images were collected. Third, the current study adopted a brain network approach compared to most previous studies in this area [30]. Studies of first language (L1) processing [35,36,37,38] and L2 processing [39,40,41,42] suggest that the functional anatomy of language processing comprises two broad processing streams: a ventral stream for speech comprehension, connecting the ventral frontal areas and temporal regions, and a dorsal stream for sensory-motor integration, involving the parietal-temporal junction and the frontal lobe. We selected ROIs to examine the interaction of the two streams as L2 proficiency increased and the possible influences of auditory perception abilities on their interactions (see Section 2 for details). 

Based on the literature reviewed above and our research design, we hypothesized that first, non-linguistic and linguistic auditory perception abilities correlate with L2 word learning attainment. Second, we predicted that these auditory perception abilities modulate the brain activations in the left IFG, auditory cortex, and parietal lobe, which are critical brain areas for L2 processing. Third, non-linguistic pitch and linguistic tone discrimination abilities may be associated with functional brain pathways that contribute to L2 learning improvement. Finally, successful learners were those individuals with better auditory perception abilities, and learning for these individuals involved more engagement in the ventral stream, which was associated with auditory perception, than in the dorsal stream, which was related to sound articulatory and auditory-motor integration.

## 2. Methods

### 2.1. Participants

Twenty-three right-handed [43] healthy college students from Pennsylvania State University participated in this study and were compensated for their time, either monetarily or with course credit, when applicable. They were participants in the learner group from Yang et al. [16]. They were all native English speakers without prior learning experience of tonal languages. All participants completed the Language History Questionnaire (LHQ 2.0, http://blclab.org/lhq2) [44], in which questions about their language history, usage habits, proficiency, and self-reported dominance were asked. The LHQ has been used in the literature as a useful tool of self-reported language background and has been found to correlate with objective assessments of language proficiency such as verbal fluency [45]. We used results from the responses to the questionnaire to exclude participants who had extensive experience with a second or third language (those who would consider themselves proficient bilingual speakers). Two participants did not complete the entire experiment, and the final sample reported here included 21 participants (10 women; mean age = 20.62 ± 1.09 years). This study was approved by the Institutional Review Board of the Pennsylvania State University and followed the research and ethics protocols used at the Penn State Social, Life, and Engineering Sciences Imaging Center.

### 2.2. Materials and Procedure

All participants completed a battery of auditory behavioral tests before the six-week L2 vocabulary training and underwent fMRI scanning following the last training session.

*Pre-training auditory behavioral tests.* To explore the role of auditory discrimination abilities in L2 learning for a tonal language, we asked participants to complete a set of auditory perception tasks, which included the discrimination of non-linguistic pitch [16], linguistic tone [46,47,48], onset [16], rhythm, and intonation [49]. In the pitch discrimination task, participants were asked to indicate whether the presented pairs of pure tones (low tone, 90 Hz vs. high tone, 100 Hz) were the same or different. During the tone discrimination task, participants heard pairs of real Chinese words (CV structure, e.g., /bi1/ and /pa2/) and judged whether the two syllables contained the same Chinese tones. In the onset discrimination task, participants judged whether the two presented real Chinese words (CV structure, e.g., /bi1/ and /pa2/) shared the same beginning consonant or not. In the rhythm discrimination, participants were presented with pairs of stimuli with varying vocalic duration of Chinese syllables and made same or different responses. Finally, in the intonation discrimination task, participants judged whether the presented pairs of stimuli with different fundamental frequency were the same or different. 

**Figure 1:**
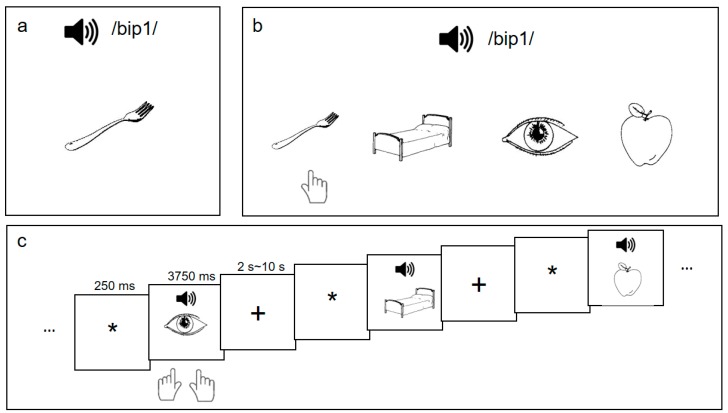
Participants were trained on 48 sound-picture associations (**a**) and were asked to complete a recognition test with feedback (**b**) after each training session. After the six-week L2 vocabulary training, they performed a sound-picture association judgment task (**c**) when their brain images were collected.

*L2 word learning procedure*. Following the pre-training behavioral tasks, participants underwent an L2 vocabulary training paradigm [16], which consisted of three training sessions per week for six weeks. In each training session, participants learned 48 sound-picture associations (henceforth the ’L2 words’): they heard a pseudo-Chinese syllable with a Mandarin tone (e.g., /bip1/) while being presented with a line-drawing picture (e.g., a fork) (Figure 1a). The 48 word stimuli were derived from a set of 16 monosyllables in the CVC (Consonant-Vowel-Consonant) structure, each superimposed with three different pitch contours of Mandarin Chinese tones (Tone 1, level; Tone 2, rising; Tone 4, falling) and the line-drawing pictures depicted familiar non-living objects corresponding to high-frequency words in English [50] and Mandarin Chinese [51].

During each 30-min training session, participants were given three presentations of the 48 sound-picture associations and were quizzed on the learned associations with feedback. After the quiz, participants reviewed the 48 pairs again in random order and completed a recognition task which assessed their L2 word-learning performance. As shown in Figure 1b, in the recognition task, the participants heard a word and judged which of the presented four pictures was the correct association of the word-sound based on their learning experience. The accuracy rate of the recognition task indicated their L2 learning achievement (L2 proficiency) after that training session.

*Post-training fMRI procedure*. Upon the completion of the training, participants performed a sound-picture association judgment task during the MRI scanning (Figure 1c). We used an event-related fMRI task with the following paradigm: after a 250-ms fixation, participants heard an auditory word for 500 ms and were presented with a picture at the same time. They judged whether the word heard and the picture matched with each other within 3750 ms (from the onset of the picture/sound). All the auditory words were among the 48 words for training and half of the words matched the pictures. The inter-trial intervals (ITI) for this task were jittered, ranging from 2 s to 10 s, with an average of 6 s. For each of the two runs, the 48-word stimuli were presented (i.e., no repetition in the same run), and comprised 24 “Yes” trials and 24 “No” trials. Participants pressed the right button with their right thumb for “Yes” responses, and the left button with their left thumb for “No” responses. 

### 2.3. MRI Acquisition

MRI images were collected on a Siemens Magnetom Trio 3T MRI scanner at the Social, Life, and Engineering Sciences Imaging Center of Pennsylvania State University, using a T2*-weighted echo-planar imaging (EPI) sequence (TE = 30 ms; TR = 2 s; flip angle = 90°; matrix size = 80 × 80; FoV = 320 mm). Participants heard auditory stimuli with MRI-compatible VisuaStim Digit headphones for auditory stimulus presentation (Resonance Technology, Northridge, CA, USA) and viewed the visual stimuli via a back-projection mirror, while their heads were immobilized with cushions. Functional images were reconstructed from 34 axial slices, with the thickness of each slice being 4 mm without a gap. To ensure tissue steady-state magnetization, we started each run with a 6-s dummy scan. High-resolution (1 × 1 × 1 mm^3^) anatomical images were acquired using a T1-weighted, 3D inversion-recovery gradient-echo (MP-RAGE) sequence. 

### 2.4. fMRI Data Analysis

*Group activation.* The fMRI data were preprocessed using SPM12 [52] and the Data Processing & Analysis of Brain Imaging (DPABI) [53] software developed in Matlab (The MathWorks Inc., Natick, MA, USA). As per the standard process, the first three scans of each participant’s dataset were discarded to allow for T1 equilibration. The remaining volumes of each run were sliced, realigned to the first volume, coregistered, normalized according to the MNI stereotactic space, resampled into 3 × 3 × 3 mm^3^ cubic voxels, and finally spatially smoothed by a 6-mm FWHM (full width at half maximum) Gaussian kernel. 

For each participant, the experimental effect (sound-picture association judgment task), with both correct trials and incorrect trials, was examined at the individual level using SPM 12. Contrast images of the experimental condition (sound-picture association judgment) and baseline condition (fixation) were entered in the second-level group analysis using a one-sample t-test to identify the group effect. Results (statistical maps) were corrected for multiple comparisons and were thresholded at *p* < 0.001 (voxel level) and family-wise error (FWE) corrected to *p* < 0.05 at the cluster-level.

*ROI Selection.* Based on the literature of functional brain networks for L1 [36,37,38] and L2 [6,39,40], we chose the following areas as ROIs in the left hemisphere: middle frontal gyrus (MFG), two sub-regions of the inferior frontal gyrus (pars opercularis, IFGop; pars triangularis, IFGtri), frontal operculum cortex (FOC), supplementary motor areas (SMA), anterior cingulate gyrus (ACC), anterior superior temporal gyrus (aSTG), posterior superior temporal gyrus (pSTG), Heschl’s gyrus (primary auditory cortex, PAC), posterior middle temporal gyrus (pMTG), posterior supramarginal gyrus (pSMG), and putamen (PU). We used DPABI to extract the time series from those 12 ROIs, anatomically defined using the Harvard Oxford Cortical and Subcortical Structural Atlas [54] for the effective connectivity analyses. Time series were also sorted into the experimental condition and the baseline condition to calculate the blood oxygen level-dependent (BOLD) signal change. These changes were correlated with behavioral auditory perception abilities.

*Effective Connectivity Analyses.* Time series from each participant were assessed using the group iterative multiple model estimation (GIMME) package [55,56,57] in R [58]. GIMME relies on the unified structural equation modeling (uSEM) and extended unified structural equation modeling (euSEM) to evaluate direct functional connectivity between ROIs at the group-level and individual-level. Specifically, uSEM examines contemporaneous and lagged (sequentially) relationships between ROIs in a blocked-fMRI study. Including lagged relationships in the model reduces statistical bias [59,60]; euSEM, based on uSEM, uses data from event-related fMRI studies to model the task and bilinear effects (i.e., how the task modulates the relationship between two nodes) after controlling for the contemporaneous and time-lagged effects among nodes. 

GIMME allows for the automated specification and estimation of group-level, subgroup-level, and individual-level relations in time series data within a structural equation model framework. First, it establishes the best model fit for each group using Lagrange Multiplier tests, which evaluate the extent to which adding a given parameter improves the model fit. Second, with the group model established, individual models are created by iteratively freeing connections until at least two of the following four modification indices indicate an excellent fit: the comparative fit index (CFI), non-normed fit index (NNFI), root mean squared error of approximation (RMSEA), and standardized root mean squared residual indices (SRMR). 

We completed the connectivity analyses using the GIMME procedure similarly as in [16], with the following criteria satisfied in the final model: CFI ≥ 0.95; NNFI ≥ 0.9. Further, we correlated the significant group-level connectivity coefficients with individual participants’ L2 proficiency (accuracy rates of sound-picture association judgment task) and auditory perception accuracy rates in order to characterize the variability in cognitive performance and the characteristics of effective connectivity among language-learning related brain regions. 

## 3. Results

### 3.1. Behavioral Results

**Figure 2:**
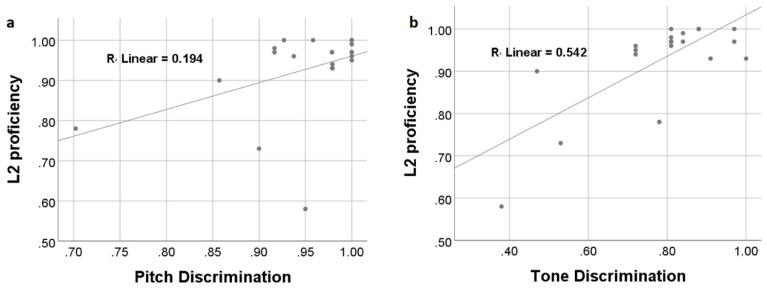
Participants’ L2 vocabulary learning performance was significantly and positively correlated with non-linguistic pitch (**a**) and linguistic tone (**b**) discrimination abilities.

After six weeks of L2 vocabulary learning, participants achieved a mean accuracy rate of 93% ± 0.11 in the sound-picture association judgment task during fMRI scanning. The accuracy rates were used to indicate participants’ L2 proficiency/attainment level. Correlation analyses showed that learners’ L2 attainment was positively and significantly correlated with their discrimination ability on the non-linguistic pitch (*r* = 0.440, *p* = 0.046) and linguistic tone (*r* = 0.736, *p* < 0.001) (Figure 2). After controlling for non-linguistic pitch perception, linguistic tone discrimination ability was still correlated with L2 proficiency (*r* = 0.695, *p* = 0.001). Linear regression analyses with both pitch and tone perception abilities entered as independent variables showed that the overall model was highly significant (adjusted-*R*^2^ = 0.537, Durbin–Watson = 1.810, *p* < 0.001).

### 3.2. fMRI Results

**Figure 3:**
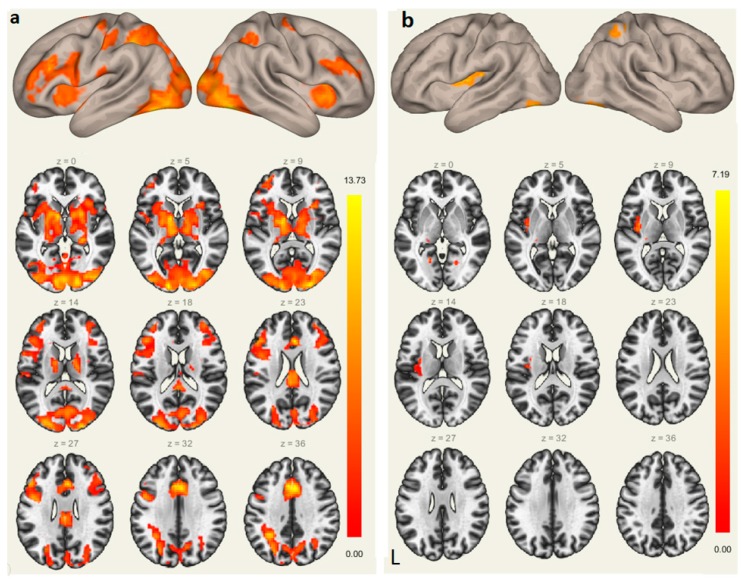
Brain activations during the sound-picture association judgment task (**a**); Brain regions where neural responses were positively related with participant’s L2 vocabulary learning attainment (accuracy rates of the sound-picture association judgment task) (**b**). L, left hemisphere.

*Brain Activations.* The sound-picture association judgment task recruited bilateral prefrontal cortex, SMA, parietal lobules, and lingual gyri. The left Heschl’s gyrus and ACC were also activated when participants recalled the learned L2 words based on the brain image contrast of the sound-picture association judgment and the fixation condition, as shown in Figure 3a. 

To explore the neural predictor of successful L2 vocabulary learning, we entered L2 proficiency as a covariate and conducted regression analysis. The results showed that L2 learning performance was significantly and positively correlated with brain activations in the following areas: the left Heschl’s gyrus (−30, −27, 12), left putamen (−18, 6, −12), right superior parietal lobule (30, −48, 54), and bilateral lingual gyri (−21, −57, −3; 21, −63, −6) (Figure 3b). 

**Figure 4:**
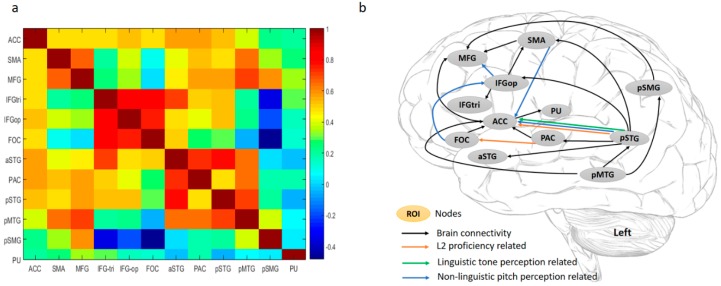
(**a**) Correlation matrix of BOLD signal changes in the regions of interest (ROIs) and (**b**) significant contemporaneous relationships between ROIs significant at the group level. ACC, anterior cingulate cortex; SMA, supplementary motor areas; MFG, middle frontal gyrus; IFGtri, inferior frontal gyrus, pars triangularis; IFGop, inferior frontal gyrus, pars opercularis; FOC, frontal operculum cortex; aSTG, anterior superior temporal gyrus; PAC, primary auditory cortex (Heschl’s gyrus); pSTG, posterior superior temporal gyrus; pMTG, posterior middle temporal gyrus; pSMG, posterior supramarginal gyrus; and PU, putamen. All regions are in the left hemisphere. A line with an arrow indicates a positive influence of one ROI on another. Lines in orange indicate that L2 proficiency is significantly and positively correlated with the connection strength of the brain pathway. Lines in green indicate that lexical tone perception ability is significantly and positively correlated with the connection strength of brain connectivity. Lines in blue indicate that non-linguistic pitch perception ability is significantly correlated with the connection strength of the brain connectivity: negative correlations for the FOC→IFGop, IFGop→MFG, and SMA→ACC pathways; positive correlation for the pSTG→ACC projection.

To examine the relationships among the ROIs, BOLD signal changes (%) of the 12 nodes were entered into a correlation analysis. As shown in Figure 4a, ACC displayed a significantly high correlation with the other nodes, except for pSMG and PU. In the frontal regions, SMA and MFG were strongly related, while IFGtri, IFGop, and FOC were highly correlated. In the temporal lobe, all the nodes were highly integrated. The pSMG displayed significant positive correlations with MFG, SMA, and pMTG, and showed a strong negative correlation with IFGtri, IFGop, and FOC in terms of brain activations. PU as a subcortical region was isolated from the other nodes in the cortical areas. In terms of frontal-temporal coactivation, the correlation matrix showed that brain activations in SMA and MFG were correlated with those in the superior and middle temporal gyri, while neural activities in IFG regions were strongly associated with those in the superior temporal regions. 

*Brain Connectivity.* As discussed in Section 2.4 of the fMRI data analysis, we used GIMME to explore directed interactions between the 12 ROIs at both lagged and contemporaneous timescales. Figure 4b shows a detailed group pattern of their contemporaneous relationships with lagged interactions controlled: during L2 word retrieval (the sound-picture association judgment task), ACC as the primary hub of this functional network exhibited the highest node degree (7 connections). It directly and positively influences MFG in the prefrontal cortex and PU in the basal ganglia, and receives information from SMA, FOC, PAC, pSTG, and pMTG. The pSTG, with a lower node degree (6 connections), is the secondary hub and located in the posterior brain, receiving a connection from the pMTG, but sends input to the SMA, IFGop, ACC, PAC, and aSTG. There are two broad streams in this network: a ventral stream and a dorsal stream. pSTG influences projections in both streams. Two pathways of the dorsal stream project from pSTG to IFGop and SMA, respectively, and finally influence MFG. The ventral pathways also begin with pSTG and meet at the ACC: one projects to the ACC directly, and the other indirectly modulates the ACC via the PAC. The pMTG influences the pSTG, connects to the MFG via the pSMG, and sends direct input to the ACC. 

*Brain-behavioral Connection.* To examine the influence of individual differences on brain activations and interactions of those ROIs, we correlated the BOLD signal changes and individual path coefficients with participants’ behavioral predictors, including L2 proficiency (the accuracy of sound-picture association judgment task), pitch (the non-linguistic pitch discrimination task), and tone (the linguistic tone discrimination task).

As depicted in Figure 4b, the non-linguistic pitch discrimination ability is negatively correlated with brain activations in the left IFGtri, IFGop, and FOC, and positively interacts with the pSMG. Behavioral-brain connectivity correlation analyses showed that (1) L2 learning attainment/proficiency was positively associated with two ventral pathways, i.e., pSTG→ACC and PAC→FOC; (2) Tone perception ability was also positively associated with the projection from the pSTG to the ACC; (3) Pitch discrimination ability negatively modulates the FOC→IFGop, IFGop→MFG, and SMA→ACC pathways, and positively influences the pSTG→ACC projection.

## 4. Discussion

The present study focused on the relationship between auditory perception abilities and tonal language learning. The results showed that first, learners’ non-linguistic pitch and linguistic tone perception performance significantly and positively predicted L2 vocabulary learning attainment. Second, L2 learning performance was associated with brain activations in the primary auditory cortex (left Heschl’s gyrus). In addition, better non-linguistic pitch perception ability was associated with decreased brain activations in the left IFGtri, IFGop, and FOC, and increased neural activity in the left pSMG. Third, within the functional network for the L2 learning, non-linguistic pitch perception ability modulated the functional brain connectivity negatively within the frontal regions (FOC→IFGop, IFGop→MFG, and SMA→ACC projections) and positively within the ventral frontal-temporal connection (pSTG→ACC). The linguistic tone perception ability, though without significant correlation with brain activations in those ROIs, positively influenced the same pSTG→ACC pathway. Finally, individuals with higher L2 attainment had better integrated ventral pathways of the frontal-temporal connection, specifically in the pSTG→ACC and the PAC→FOC connection pathways. This result is consistent with our hypothesis that better auditory perception abilities are associated with increased functional connectivity in the pSTG→ACC ventral pathway, implying that auditory perception ability contributes to L2 learning. 

### 4.1. L2 Word Learning Depends on Auditory Perception Abilities in the Processing of Auditory Decoding, Articulatory Rehearsal, and Phonological Storage 

L2 word learning involves auditory decoding, articulatory rehearsal, phonological storage, and lexical retrieval under domain-general control. Our findings suggest the following aspects in which L2 word learning may be influenced by auditory abilities. 

First, with better auditory perception abilities, auditory encoding is more successful and efficient. Therefore, the present study showed a significant correlation between L2 attainment and brain activations in the primary auditory cortex (left Heschl’s gyrus). The left Heschl’s gyrus is typically associated with auditory processing in non-speech domains but has also been implicated as an important region for speech processing. Consistent with the data reported here, Wong et al. [61] showed that, in a similar tonal-language word-training study, less successful learners had smaller brain volume in the left Heschl’s gyrus. These results suggest that the left Heschl’s gyrus is essential for encoding acoustic cues during spoken language learning. 

As shown in Figure 4a, the PAC (left Heschl’s gyrus) is highly integrated with the aSTG, pSTG, and pMTG. The pSTG has been implicated as a hub for speech comprehension, key for accessing lexical phonology [62] or extracting phonetic information of acoustic signals, especially the perception of phonetic categories [63]. Unlike the PAC and pSTG in speech encoding, the aSTG and pMTG are more involved in linguistic, particularly semantic processes: for example, aSTG is associated with the semantic storage of learned words [64], and pMTG is part of the semantic control network [65].

Second, better auditory perception abilities free the cognitive demand on the sound articulatory mechanism for rehearsal and reduce the competition for lexical selection in the frontal regions. Findings from this study indicate that better non-linguistic pitch perception is associated with decreased brain activations in the IFGop, IFGtri, and FOC. Previous studies have shown that L2 learning is related with increased brain volume or activations in the left IFG and SMA [13,21,66,67], indicating effortful lexical retrieval or articulatory planning that may contribute to the consolidation of L2 phonetic representations. Our findings suggest that increased auditory encoding abilities reduce the efforts to articulate and integrate sound-motor information. The FOC has been suggested to mediate syntactic processes during auditory language comprehension [68]. Although our current study does not involve syntactic processing or learning, better auditory perception abilities may enable learners to identify the syntactic category of words that are heard.

Third, although our focus in this study is on the relationship between auditory perception ability and L2 learning attainment, we performed one additional analysis to identify whether auditory ability would also predict learning improvement. Our participants had no way of knowing the Chinese pseudowords before training; therefore, their recognition accuracy rates at each training session were their degree of improvement. To this end, we correlated the recognition accuracy of the first and the last training session as well as their differences (i.e., improvement between session 1 and session 18) with the auditory perception ability scores. Although we found that neither pitch or tone perception correlated with their differences, participants’ learning performance at both sessions was significantly and positively correlated with tone discrimination ability (1st session, *r* = 0.617; 18th session, *r* = 0.597), but not with the pitch discrimination performance. This correlation indicates that auditory ability, at least tone discrimination, predicts not only learning success, but also learning improvement at different stages.

Finally, better auditory perception abilities improve the quality of phonological representations of lexical items. Our study found that increased non-linguistic pitch discrimination performance is associated with increased brain activations in the left pSMG, which is well-known for the storage of learned words in L2 learning [69,70]. It is possible that individuals with better auditory perception have more accurate information about the target lexical item, which was highly activated, reflected by increased neural responses in the pSMG in the present work.

### 4.2. L2 Word Learning Success Lies in the Collaboration of Dorsal and Ventral Streams of the L2 Language Network

Studies on brain structural connectivity indicate the importance of frontal-temporal connection for L2 learning. Wong et al. [71], using diffusion tensor imaging (DTI), showed that sound-to-word learning performance is correlated with white matter anisotropy in the left parietal-temporal region which belongs to a ventral stream in the language network. Schlegel et al. [72] found increased frontal-temporal connectivity in the left hemisphere and increased connectivity between left-hemisphere language regions and their right homologue. Xiang et al. [73] further tested students who learned Dutch in a 6-week course. They found that with increasing L2 proficiency, the frontal-temporal pathway shifts from the left to the right hemisphere and with further increased proficiency, this shift is set back to the original state. These findings suggest structural brain changes with L2 proficiency increases.

Studies on the functional brain connectivity such as ours here provide complementary evidence to these structural imaging data mentioned above. For example, Veroude et al. [14] reported stronger connectivity between the left and right SMG only for successful learners. Yang et al. [16] revealed more frontal-temporal connections in the successful learners compared to less successful learners of Chinese words, and Grant et al. [19], in a Spanish learning study, showed that L2 proficiency increases as frontal centered control network shifts to diverse frontal and temporal networks that engage more efficient automatic semantic processing.

The present study highlights the importance of the frontal-temporal connections under the supervision of the ACC, a key attention and conflict monitoring center [44,74,75]. Based on previous language models [36,37,38] and the findings of the current study, we suggest that L2 word-learning success depends on the collaboration of dorsal and ventral streams. As shown in Figure 4b, the ventral stream of the language network serves the linguistic processing of novel words in the L2 learning context: pSTG→PAC and pSTG→aSTG are responsible for auditory perception; PAC→FOC reflect that the acoustic information is sent to FOC for syntactic analysis of auditory sequence; pMTG→pSTG implies semantic control on the speech perception; pSTG→ACC, PAC→ACC, PAC→FOC→ACC, and pMTG→ACC all reflect feedback on auditory perception to the monitoring system ACC.

In contrast, the dorsal stream serves the purpose of sound-articulatory mapping and lexical selection (identification): pSTG→SMA and pSTG→IFGop are responsible for the articulation of speech being heard; IFGop→IFGtri is associated with lexical information selection, which reports to MFG for evaluation [76]; and pMTG retrieves lexical knowledge from pSMG, which will be less demanding with better quality of lexical representation as a result of better auditory perception abilities. 

As L2 proficiency increases, the pSTG→ACC, PAC→FOC, two projections of the ventral stream, might be strengthened for better integration of frontal and temporal regions, which represents the neural adaptation in the successful L2 learners. Our findings that the ventral stream is the major language pathway in L2 processing are consistent with findings from native language studies [77] where the dorsal pathway connecting the STG and the premotor areas serves sublexical repetition and the ventral pathway connecting the MTG and the ventrolateral prefrontal cortex mediates linguistic processing of sound to meaning, which is the major language pathway of native language processing. 

The current study also shows that individuals with better auditory perception abilities, like successful learners, increase the engagement of the ventral pathway for efficient L2 lexical comprehension and have less demand on the local connections within the frontal areas, and the dorsal pathway assists the ventral pathway in the semantic selection and articulatory rehearsal. Individuals with higher L2 attainment and those with better non-linguistic pitch or linguistic tone perception abilities both have strengthened pSTG→ACC ventral projection, implying that auditory perception and the feedback from the auditory perception center to the domain-general monitoring center is vital for L2 learning success.

## 5. Conclusions and Limitations

In conclusion, the current study reveals the neurocognitive mechanisms underlying auditory perception in L2 word learning. We found that L2 learning depends on the collaboration of a ventral stream for speech perception and a dorsal stream for sensory-motor mapping in the left hemisphere. By tracing the interactions between language areas in a network and examining brain-behavioral correlations, our approach allows for an integrative and neurocognitively informed understanding of behavioral and biological predictors of L2 learning. Our study indicates the distinct contribution of the ventral and dorsal stream within the L2 network. With better auditory perception abilities, the ventral pathway in the frontal-temporal regions is strengthened as L2 proficiency increases, consistent with findings from previous work using brain network analyses [16,19]. In L2 learning, successful learners have strengthened ventral pathways, implying that the ventral stream plays a more dominant role than the dorsal stream, as the former serves a speech perception function and the latter is associated with sensory-motor mapping. Better auditory perception abilities before learning also predict higher L2 proficiency and are associated with the stronger engagement of the ventral pathways during the retrieval of the learned vocabulary.

One limitation of the current study is that our study did not test participants with an auditory screening test such as the pure tone audiometry task [78], which might further inform the relationship of auditory perception abilities and vocabulary learning. Additional studies should consider this in the future. Another limitation is, like most studies on language networks [36,38,79], that our study focused on the connectivity patterns only in the left hemisphere as we wanted to limit the number of ROIs in the analyses. Given recent work, as discussed above, that indicates roles of both hemispheres in L2 learning [7,28,72,73], it would be of interest to examine both hemispheres and address whether the recruitment of the ventral and dorsal streams will change as L2 learning progresses. Future studies should pursue large-scale connectivity analyses to better interpret the joint and distinct roles of the dorsal and ventral pathways for L2 learning.
